# The Effects on Sensorial Block, Motor Block, and Haemodynamics of Levobupivacaine at Different Temperatures Applied in the Subarachnoid Space

**DOI:** 10.1155/2014/132687

**Published:** 2014-03-18

**Authors:** Bahittin Nazli, Huseyin Oguzalp, Eyup Horasanli, Mehmet Gamli, Beyazit Dikmen, Nermin Gogus

**Affiliations:** ^1^Department of Anesthesiology, Turan Turan Hospital, 16140 Bursa, Turkey; ^2^Department of Anesthesiology, Medicabil Hospital, 16140 Bursa, Turkey; ^3^Department of Anesthesiology and Intensive Care, Health Ministry Kecioren Education and Research Hospital, 06280 Ankara, Turkey; ^4^Department of Anesthesiology and Intensive Care, Health Ministry Numune Education and Research Hospital, 06230 Ankara, Turkey; ^5^Department of Anesthesiology and Intensive Care, Gazi University Faculty of Medicine, 06560 Ankara, Turkey

## Abstract

*Aim*. To evaluate the effects of 0.5% levobupivacaine at 37°C preheated from room temperature, on sensorial block, motor block, and haemodynamics in patients undergoing transurethral prostate resection (TUR-P). *Material and Method*. The patients were randomly allocated to two groups: Group I patients were injected with 3 mL 0.5% levobupivacaine solution which had been kept at room temperature for at least 24 hours and Group II patients were injected with 3 mL 0.5% levobupivacaine solution which had been kept at 37°C for at least 24 hours. The patients were examined in terms of sensorial block, motor block, haemodynamic profile, and incidence of side effects. *Results*. No significant difference was found between the groups in terms of demographic data. The time to reach *T*_10_ sensory block and the time of starting motor block were found to be significantly shorter in Group II . The duration of sensory block over *T*_10_ and *T*_6_, the duration of *L*_1_ regression, the duration of the sensory block, and the regression time of the motor blocks from 3 to 2 were found to be longer in Group II. *Conclusion*. The use of 0.5% levobupivacaine spinal anaesthesia heated to 37°C accelerated the start of sensory and motor block.

## 1. Introduction

Although there are in vitro studies showing changes in the density of local anaesthetic (LA) at different temperatures [1–6], there are a limited number of studies showing the effects of these changes in density from different temperatures on spinal anaesthesia clinical results [7–9].

The decrease in density in inverse proportion to the increase of the liquid temperature was explained by Davis and King [10] giving examples of the relationship between temperature and density (Table 1).

A curvilinear reduction was seen in the density with increased temperature of the LA solution. Changes occurring in density with reflected in the baricity [1–5, 11]. Injections of room temperature LA solutions into body temperature cerebrospinal liquid (CSF) cause an immediate local reduction in CSF temperature (2-3°C with 2.4 mL bolus; 6–8°C with 12 mL bolus) but the CSF returns to normal temperature within 2 min. This happens before spinal root fixation of the local anaesthetics [1–3]. Therefore when there is synchronisation of the temperature, the local anaesthetic solution in the CSF will display a mild hyperbaric property and thus the position of the patient will affect the distribution of the local anaesthetic.

Results from the effect of temperature are related more to the use of solutions without additives. For example, in a study by McLeod using a mechanical oscillation resonance method to investigate the relationship between temperature and density, it was determined that 37°C densities (0,99944 for 0.5% bupivacaine, 1,00024 for 0.5% levobupivacaine, and 0,99953 mg/mL for 0.5% ropivacaine) were lower than 23°C densities (1,00376 for 0.5% bupivacaine, 1,00419 for 0.5% levobupivacaine, and 1,00380 mg/mL for 0.5% ropivacaine) [4]. With 7% dextrose, the 25°C and 37°C densities of 0.5% bupivacaine were equal (1,028 g/cm^3^) [9].

The temperature of local anaesthetics has an effect on *pK*_*a*_ values. Increased temperature of local anaesthetics by decreasing *pK*_*a*_ approaches the physiological pH and causes an increase in the fraction of nonionised local anaesthetic. This speeds up the start of the effect, increases the quality of the block, and lengthens the duration of the block [12, 13]. While *pK*_*a*_ is 7.92 in lidocaine at 25°C, at 40°C it is 7.57 [14]. At 10°C, the *pK*_*a*_ value of bupivacaine is 8.49 and at 38°C, it is 7.92. The *pK*_*a*_ value of mepivacaine at 10°C is 8.02 and at 38°C it falls to 7.55 [15].

On a thermodynamic basis, when increased temperature increases molecular kinetic energy, the number of moving particles increased [16]. Thus increased temperature causes increased molecular activity of the local anaesthetic solution and facilitates distribution in the CSF and higher levels of spinal anaesthesia are reached [9].

On the other hand, levobupivacaine has less affinity and strength of depressant effects on to myocardial and central nervous vital centers and a superior pharmacokinetic profile. Clinically, levobupivacaine is well tolerated in a variety of regional anesthesia.

In the current study, using a 0.5% levobupivacaine solution kept at either room temperature (20–24°C) or body temperature (36–37°C) for 24 hours and then injected into the subarachnoid space in patients undergoing TUR-P for BPH, it was aimed to compare the effects of this temperature difference on spinal anaesthesia characteristics from motor and sensory block starting time, maximum block level, duration of the block, and haemodynamic parameters.

## 2. Method

Approval to the study was granted by the Local Ethics Committee and informed consent was obtained from all the study participants. The study comprised 60 patients with BPH in the ASA I-III risk group, aged 18–75 years, for whom elective TUR-P was planned. Any patients with a neurological deficit, allergy to local anaesthetic, or with contraindications for spinal anaesthesia were excluded from the study.

Before anesthesia induction, standard monitoring was applied to the patients of electrocardiography, and non-invasive measurements of arterial pressure (systolic, diastolic, mean), heart rate and oxygen saturation with monitor (Drager, Julian Plus Vitara 8060, Germany). Conditions were provided, so that a transfer to general anaesthesia could be made at any moment.

Patients were allocated randomly to the two groups. A venous route on the back of the hand was opened with a 20 gauge iv cannula in a peripheral vein. Following the start of replacement with 7–10 mL/kg 0.9% saline, the patients were put into a sitting position. In accordance with asepsis and antisepsis regulations, the subarachnoid space was entered with a 25 gauge Quincke spinal needle from the* L*_3_-*L*_4_ interspinous space. When CSF flow was seen, the spinal needle opening was turned caudally.

The 30 patients in Group I were injected with 3 mL 0.5% levobupivacaine solution (chirocaine 0.5%, Abbott Laboratories, Istanbul) which had been kept at room temperature (mean 23°C) for at least 24 hours and the 30 patients in Group II were injected with 3 mL 0.5% levobupivacaine solution which had been kept at 37°C for at least 24 hours. All injections were made at the rate of 0.2 mL/sec.

To keep the levobupivacaine flacons at 37°C, a bain-marie set to 37°C was used (Elektro-mag GEMO Temperature Controller DT 109).

After the LA solution into the subarachnoid space, the patients were lie down and the administration of 2 lt/min O_2_ was started with a face mask. When it was determined that a sufficient level of block (*T*_10_ dermatome and above) had been reached, the patient was put into the lithotomy position and the surgery was allowed to start. In cases where the sensory block did not reach* T*_10_, it was decided to administer general anaesthesia.

From the moment, the patient entered the operating theatre and throughout the operation a record was made of systolic, diastolic, and mean blood pressure, heart rate, and SpO_2_ (at 5 min intervals for the first 30 min, at 10 min intervals for the next 30 mins, then at 15 min intervals up to 90 mins, and at 30 min intervals thereafter).

Patients were monitored for any side effects of nausea, vomiting, bradycardia, hypotension, and reduced SpO_2_ (<93%). Throughout the monitoring, if systolic blood pressure was determined to have dropped by more than 30% of the preoperative basal values, a rapid iv infusion of 0.9% saline was administered and if necessary a 10 mg iv bolus of ephedrine at 1 min intervals and if heart rate fell below 50/min, a 0.5 mg iv bolus of atropine was administered. If nausea and vomiting were determined, metoclopramide was given as 10 mg iv and a fall of SpO_2_ below 93% was evaluated as hypoxia and 4 lt/min^−1^ oxygen was administered via a face mask.

The levels of sensory block and motor block were recorded by evaluations at 5 min intervals for the first 30 min, at 10 min intervals for the next 30 mins, then at 15 min intervals up to 90 min, and at 30 min intervals thereafter.

The sensory block level was defined as the dermatome where the sensory response was lost with the pinprick test on the bilateral anterior axillary line.

*The time taken for the sensory block to reach T*_10_; the time taken for the sensory block to reach the* T*_10_ dermatome from the injection of the LA solution into the subarachnoid space;* maximum sensory block level*; the highest level dermatome where the sensory response was lost with the pinprick test;* the time to reach maximum sensory block*; the time taken to reach the highest sensory block level from the injection of the LA solution into the subarachnoid space;* the duration of the sensory block at T*_10_ *and above*; the total time that there was no sensory response with the pinprick test at* T*_10_ and above;* the duration of the sensory block at T*_6_ *and above*; the total time that there was no sensory response with the pinprick test at* T*_6_ and above;* 2 segment regression time*; the mean time taken for the sensory block to reduce to two dermatomes below the highest level;* L*_1_ *regression time*; the mean time taken for the distribution ensory block to fall from the highest level to the* L*_1_ dermatome level. The mean times of the rising sensory block with the LA injection and the fall to* L*_1_ dermatome were evaluated and recorded.

The motor block was evaluated with the Modified Bromage Score (0 =* no paralysis the patient can fully flex the foot and knee*, 1 =* the patient cannot raise a straight leg, the knee and foot can be moved*, 2 =* the knee cannot be brought to flexion, only the foot can be moved*, 3 =* foot joints or toes cannot be moved, total paralysis*).

The* 10-minute Bromage score* was evaluated and recorded as the degree of motor block 10 minutes after the LA injection into the subarachnoid space.* The time of starting motor block*; the time taken from the LA injection into the subarachnoid space for full motor block (Bromage score 3) to form in the lower extremities.* Bromage score 3 to 2 regression time*; the time taken from full motor block of the lower extremities to a return of the ability to move the feet.

At the end of surgery, the surgeon evaluated the ease of the operation as poor (0), moderate (1), or good (2).

An evaluation was requested from the patient postoperatively in the form of (1) I would not prefer this type of anaesthesia in the future or (2) I would prefer this type of anaesthesia in the future.

### 2.1. Satatistical Analysis

Data analysis was made with statistical package for social science ( SPSS ) 11.5 package programme. It was decided to take at least 12 subjects for each group. The distribution of the data obtained from the measurements was examined with the Shapiro Wilk test for conformity to normal distribution. The features of the descriptive statistics obtained from the measurements are given as mean ± standard deviation or mean (minimum-maximum) and the categorical variables are shown as number of cases and percentages (%).

Whether there was any statistically significant difference between the groups in normally distributed continuous variables was examined with Student's *t*-test and with the Mann Whitney *U* test for nonnormal distibution continuous variables.

Repeated measures variance analysis was used to evaluate any statistically significant difference in repeated measurements in the groups. Where the statistical result of the repeated measures variance analysis was found to be significant, the Bonferroni correction multiple comparisons test was used to determine the reason for the difference at the time of measurement.

For categoric comparisons, Chi-Square or Fisher's Exact probability test was used. A value of *P* < 0.05 was accepted as statistically significant for all the results.

## 3. Results 

The demographic characteristics (age, weight, andheight) of the patients, ASA classifications and duration of surgery are given in Table 2. There was no statistically significant difference between the groups.

Sensory block of* T*_10_ or above was reached as the criteria for surgery to commence for all patients. The duration of the sensory block at* T*_10_ or above was 97.33 ± 26.31 min for Group I which was shorter than the 140.57 ± 22.30 min in Group II. The difference between the groups was found to be statistically significant (*P* < 0.001).

The difference between the groups in respect of the duration of the sensory block was found to be statistically significant (*P* = 0.004) (Table 3). The distributions of maximum sensory block levels according to the groups are shown in Table 4.

**Figure 1:**
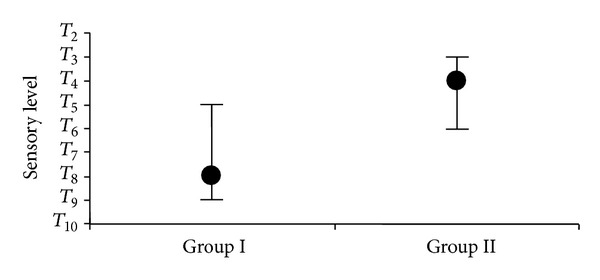
Maximum sensory block levels; median (max-min).

While the median level of the maximum sensory block level in Group I was* T*_8_ (mean ± SD; 7.86 ± 1.07), it was* T*_4_ in Group II (mean ± SD; 4.16 ± 0.91) (Figure 1). The difference between the groups was found to be statistically significant (*P* < 0.001).

**Figure 2:**
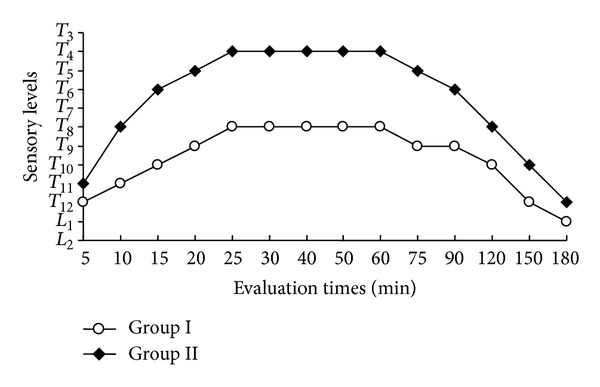
Sensory block levels according to evaluation times.

The sensory block levels of the groups according to the time of evaluation are shown in Figure 2. At each evaluation time, the sensory block levels of Group I were found to be lower than those of Group II. At all the evaluation times, the differences between the groups in the sensory block levels were statistically significant (*P* < 0.001 for 5 min–150 min; *P* < 0.05 for 180 min).

**Figure 3:**
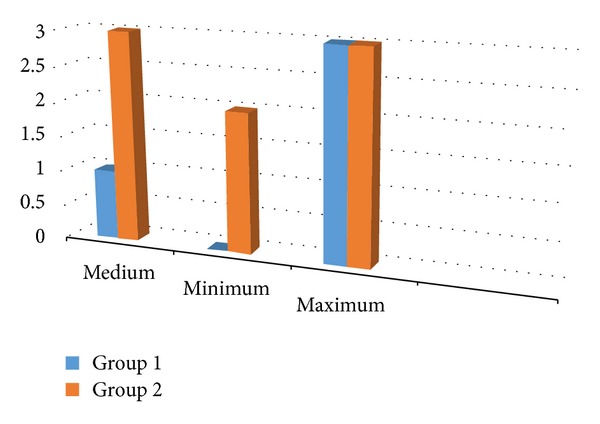
10-minute Bromage scores.

The 10-minute Bromage score was 1 in Group I and 3 in Group II (Figure 3). The difference was statistically significant (*P* < 0.001).

Motor block started at 18.23 ± 5.27 min in Group I and at 11.43 ± 3.52 min in Group II. The difference between the groups was found to be statistically significant (*P* < 0.001).

The time of regression of the Bromage score from 3 to 2 was 142.17 ± 28.03 min in Group I and 156.83 ± 32.60 min in Group II. The difference between the groups was found to be statistically significant (*P* = 0.049 < 0.05).

When the mean HR measurements obtained throughout the monitoring were compared, there was no statistically significant difference between the groups (*P* = 0.818 > 0.05).

A statistically significant difference was determined between the repeated HR measurements in Group I (*P* < 0.001). The statistically significant decrease in mean HR in this group started from 25 min (*P* < 0.01). In Group II, a statistically significant difference was determined between the repeated HR measurements (*P* = 0.003). In this group, the statistically significant decrease in mean HR in this group started from 20 min (*P* < 0.05).

When the mean systolic blood pressure (SBP) measurements obtained throughout the monitoring were compared, a statistically significant difference was determined between the groups (*P* = 0.017). In general, the SBP level of Group I was higher than that of Group II.

When the mean SBP values of the groups were compared according to the time of evaluation, no statistically significant difference was determined in the preblock values (*P* = 0.436). A statistically significant reduction was determined between the repeated SBP measurements in Group I (*P* = 0.003). Within this group, a statistically significant reduction occurred in the mean SBP from 40 min on *P* < 0.01. A statistically significant reduction was determined between the repeated SBP measurements in Group II (*P* < 0.001). Within this group, a statistically significant reduction occurred in the mean SBP from 10 min on *P* < 0.01.

When the mean diastolic blood pressure (DBP) measurements obtained throughout the monitoring were compared, a statistically significant difference was determined between the groups (*P* = 0.048). In general, the SBP level of Group I was higher than that of Group II.

When the mean SBP values of the groups were compared according to the time of evaluation, no statistically significant difference was determined in the preblock values (*P* = 0.532).

A statistically significant reduction was determined between the repeated DBP measurements in Group I (*P* = 0.043). Within this group a statistically significant reduction occurred in the mean DBP from 30 min on *P* < 0.01. A statistically significant reduction was determined between the repeated DBP measurements in Group II (*P* < 0.001). Within this group, a statistically significant reduction occurred in the mean DBP from 5 min on *P* < 0.05.

When the mean blood pressure (MBP) measurements obtained throughout the monitoring were compared, a statistically significant difference was determined between the groups (*P* = 0.047). In general, the MBP level of Group I was higher than that of Group II.

When the mean MBP values of the groups were compared according to the time of evaluation, no statistically significant difference was determined in the preblock values (*P* = 0.771).

A statistically significant reduction was determined between the repeated MBP measurements in Group I (*P* = 0.006). Within this group, a statistically significant reduction occurred in the mean MBP from 75 min on *P* = 0.043. A statistically significant reduction was determined between the repeated MBP measurements in Group II (*P* < 0.001). Within this group a statistically significant reduction occurred in the mean MBP from 5 min on *P* < 0.05.

No statistically significant difference was determined between the groups in the saturation (SpO_2_) measurements obtained throughout the monitoring period (*P* = 0.235 > 0.05).

There was similar prevalence of hypotension, bradycardia, nausea, and vomiting between the groups (*P* values: 0.112, 0.554, 1.000, and 1.000, resp.). Comparison was not made for these side effects in any case where there was no reduction in saturation level (SpO_2_ < 93%).

While there was no need for ephedrine in Group I, when 4 patients in Group II required the administration of ephedrine, a reduction in SBP values was determined. The difference between the group was not found to be statistically significant (*P* = 0.112).

In 2 patients in Group I and 1 patient in Group II, when there was a need for the administration of atropine, HR was determined to have dropped below 50/min. The difference between the group was not found to be statistically significant (*P* = 1.000).

There was no difference between the groups in terms of patient and surgeon satisfaction levels (*P* = 1.000).

## 4. Discussion

Various factors have been reported to affect the intrathecal distribution of local anaesthetic solutions [17, 18]. Features such as the concentration of the injected solution, volume, baricity, density, and temperature are important amongst these factors [11, 17].

In a search of literature, no in vivo studies were found which researched the effects on clinical results of 0.5% levobupivacaine at different temperatures on spinal anaesthesia distribution. However, in vivo and in vitro studies conducted on nerve blocks have shown levobupivacaine to be as powerful as bupivacaine and provides similar sensory and motor block [19–21].

According to Richardson and Wissler [22], the upper level of hypobaricity in males is 1.00028 g/ml and hyperbaricity lower level is 1.00100 g/mL [23]. When the measurements of this researcher are used, 0.5% levobupivacaine solution is mildly hyperbaric at 23°C (density: 1.00419 (0.00002) mg/mL) and mildly hypobaric at 37°C (density: 1.00024 (0.00009) mg/mL).

In the current study, from the intrathecal application in a sitting position of 3 mL of 0.5% levobupivacaine solution at different temperatures (37°C and room temperature), the mean maximum sensory block levels of the 37°C and room temperature groups were found to be* T*_4_, 16 ± 0.91 (*T*_3_–*T*_6_) and* T*_7_, 86 ± 1.07 (*T*_5_–*T*_9_), respectively. This difference in the mean maximum sensory block levels was statistically significant. All the sensory block levels of the evaluation times in Group II were statistically significantly high compared to Group I.

When compared with CSF density, that 0.5% levobupivacaine solution is mildly hyperbaric at 37°C and mildly hypobaric at room temperature [22] may explain the higher maximum sensory block levels obtained in Group II. On a thermodynamic basis, the increased temperature of levobupivacaine increases molecular kinetic energy and thereby the number of active particles which is also considered to have a possible contribution to the higher sensory block levels.

In the current study, the standard deviation (SD) values of the mean maximum sensory block levels in Group I and Group II were ±0.91 ve ±1.07 and minimum-maximum values were* T*_6_–*T*_3_ and* T*_9_–*T*_5_, respectively. It is thought that the SD values of Group II being lower than those of Group I and the minimum-maximum values being close to each other is because the maximum sensory block level may be more easily predicted.

When the mean highest sensory block levels are considered (Group II,* T*_4_, 16; Group I,* T*_7_, 86), it is seen that the time per segment to reach these levels was shorter in the 37°C group. In addition, the time taken to reach the sensory block level of* T*_10_ in Group II determined in this study was statistically significantly shorter compared to Group I (13.86 ± 3.73). These results show that heating levobupivacaine to 37°C increased the speed of the sensory block. This result can be explained by the temperature increase lowering the density and thereby the baricity. Also, by lowering the *pK*_*a*_ value of increased temperature local anaesthetics, the physiological pH is approached [12–15] and it is thought that the start of the effect is accelerated by increased non-ionised fraction resulting from the reduced *pK*_*a*_ created in 37°C levobupivacaine.

In the current study, the 2 segment regression time for 0.5% levobupivacaine was determined as shorter in Group II (69.40 ± 17.13 min) compared to Group I (77.80 ± 23.40 mins). No statistically significant difference was determined in this difference of 2 segment regression. It is thought that the shorter time of 2 segment regression in Group II can be explained by greater distribution within the CSF of the local anaesthetic solution at this temperature and because of this lower concentration, the regression was accelerated.

Another means of evaluating sensory block regression is to determine the time of the sensory block at a specified dermatome level. In the current study, the mean time of the 0.5% levobupivacaine sensory block above* T*_6_ and* T*_10_ was found to be 80.00 ± 23.92 min and 140.57 ± 22.30 min, respectively, in Group II and 5.00 ± 14.14 min and 97.33 ± 26.31 min, respectively, in Group I. The difference in these times was found to be statistically significantly longer in Group II.

It is thought that the shorter times of the sensory block at these levels may be due to 0.5% levobupivacaine at room temperature being mildly hyperbaric and the low number of patients in whom the sensory block was able to exceed* T*_10_ and* T*_6_ segments from the intrathecal application in a sitting position.

In the current study, the duration of* L*_1_ regression was determined as 167.73 ± 23.48 min in Group II and 148.13 ± 24.77 min in Group I with Group II being statistically significantly longer. In addition, the duration of the 0.5% levobupivacaine sensory block was determined as statistically significantly longer in Group II (190.00 ± 24.63 mins) than in Group I (172.50 ± 24.16 mins). Taking the mean highest sensory block levels into consideration, when the number of segments involved had been compared, more segments had been seen to be involved in Group II and the sensory block regression times per segment had been shorter. The heating of 0.5% levobupivacaine to 37°C provided a higher sensory block which lasted longer.

In conclusion, it can be said that the use of 0.5% levobupivacaine solution heated to 37°C not only provides a higher level of sensory block of a more predictable level, even though the regression time per segment is shorter, but also a longer-lasting sensory block. Given the times obtained in this study to reach the* T*_10_ level of the sensory block, a more rapid start to the moror block is a result which can be expected.

The time taken to the start of the motor block in this study was determined as mean 11.43 ± 3.52 min in Group II and 18.23 ± 5.27 min in Group I. In addition the 10-minute Bromage score was found to be 3 in Group II and 1 in Group I. These differences between the groups were statistically significant. The time of the Bromage score regression from 3 to 2 was determined as 156.83 ± 32.60 min in Group II and 142.17 ± 28.03 min in Group I. The difference between the groups was found to be statistically significant.

As 0.5% levobupivacaine at 37°C became mildly hypobaric, it prolonged sensory block regression and motor block regression and this change in the baricity is thought to arise more from the spread of cephalin than the block.

In terms of haemodynamic changes in the current study, a reduction of a statistically significant level was determined in Group II compared to Group I. The reductions seen in the blood pressures of Group II can be explained by this group more rapidly reaching the block and having higher levels of sensory block. The drop in blood pressure values in both groups compared to the baseline values is thought to be associated with the drop in peripheral vascular resistance with spinal anaesthesia.

No statistically significant difference was determined between the groups in terms of saturation (SpO_2_) measurements. The SpO_2_ values did not fall below 93% in any case of the current study. This is thought to have been affected by the administration of oxygen via a face mask following the intrathecal application of local anaesthetic solution to the patients in the current study. Manara et al. emphasised the need for oxygen support for patients using sedative medication during routine spinal anaesthesia [23].

No statistically significant difference was determined between the groups of the current study in terms of the side effects of bradycardia, nausea, and vomiting. While a fall in SBP was determined in 4 patients of Group II who required ephedrine, no patient required ephedrine in Group I. This may be explained by Group II having reached higher levels of sensory block. In 1 patient of Group II who required atropine, a fall in HR was determined and in 2 patients of Group I who required atropine the fall in HR was determined as 48/min. The histories of the room temperature group patients who received atropine revealed preoperative HR measurements of approximately 50–55/min. No statistically significant difference was determined between the groups in respect of ephedrine and atropine use.

No difference was determined between the groups in this study in terms of patient and patient and surgeon satisfaction.

Similar results have been obtained to those of the current study; the effects on sensory block, motor block, and haemodynamics of subarachnoid space application of bupivacaine solution at different temperatures [7–9, 18]. In vitro studies showing the effects on density of different temperatures of local anaesthetic solution have features supporting the results obtained in the current study [1–6].

## 5. Conclusion

Time necessary for synchronisation of temperature within the CSF, the temperature of 0.5% levobupivacaine solution, is a significant factor in the determination of the sensory spread. It is easy to predict the analgesia levels when levobupivacaine solution preheated to 37°C is used. When a high level, long-lasting sensory block is required, the use of 0.5% levobupivacaine solution heated to 37°C is an attractive alternative method.

## Figures and Tables

**Table 1:** The effect of heat on liquid density.

Temp. (°C)	Density (gr/mL)	Difference (gr/mL)
4	1.000	
15	0.9991	0.0009
25	0.9971	0.0020
37	0.9934	0.0037

**Table 2:** Demographic data, ASA classification, and duration of operation for all the patients.

	Group I (room temperature) *n* = 30	Group II (37 °C) *n* = 30	*P* value
Ages (years)	65.90 ± 10.72	65.57 ± 7.44	0.889
Weight (Kg)	70.83 ± 9.90	71.50 ± 11.60	0.812
Height (cm)	169.37 ± 4.97	169.10 ± 5.44	0.844
ASA I/II/III	2/23/5	1/26/3	0.598
Duration of operation (min)	43.23 ± 10.31	45.90 ± 13.14	0.386

Values are given as mean ± standard deviation (mean ± SD).

**Table 3:** Comparison of the sensory block data.

	Group I (room temp.) *n* = 30	Group II (37 °C) *n* = 30	*P* value
Time to reach *T*_10_ (min)	13.86 ± 3.73	6.40 ± 1.61	0.000*
Time to reach maximum sensory block (min)	24.37 ± 5.90	22.27 ± 4.27	0.178
Duration of sensory block at or above *T*_10_ (min)	97.33 ± 26.31	140.57 ± 22.30	0.000*
Duration of sensory block at or above *T*_6_ (min)	5.00 ± 14.14	80.00 ± 23.92	0.000*
Duration of 2 segment regression (min)	77.80 ± 23.40	69.40 ± 17.13	0.084
Duration of *L*_1_ regression (min)	148.13 ± 24.77	167.73 ± 23.48	0.003*
Duration of sensory block (min)	172.50 ± 24.16	190.00 ± 24.63	0.004*

Values are given as mean ± standard deviation (mean ± SD).

*Statistical significance of difference between mean values (*P* < 0.05).

**Table 4:** Distribution of maximum sensory block levels according to the groups.

Group	Maximum level of sensory block							Total
	*T*_3_	*T*_4_	*T*_5_	*T*_6_	*T*_7_	*T*_8_	*T*_9_	
I (room temp.)								
Number	0	0	1	3	4	13	9	30
%	0	0	3.3	10	13.3	43.3	30.0	100.00
II (37 °C)								
Number	7	14	6	3	0	0	0	30
%	23.3	46.7	20	10	0	0	0	100.00

Total								
Number	7	14	7	6	4	13	9	60
%	11.7	23.3	11.7	10	6.7	21.7	15	100.00

## References

[1] Horlocker TT, Wedel DJ (1993). Density, specific gravity, and baricity of spinal anesthetic solutions at body temperature. *Anesthesia and Analgesia*.

[2] Davis H, King WR (1952). Densities of common spinal anesthetic solutions at body temperature. *Anesthesiology*.

[3] Ernst EA (1968). In-vitro changes of osmolality and density of spinal anesthetic solutions. *Anesthesiology*.

[4] McLeod GA (2004). Density of spinal anaesthetic solutions of bupivacaine, levobupivacaine, and ropivacaine with and without dextrose. *British Journal of Anaesthesia*.

[5] Heller AR, Zimmermann K, Seele K, Rössel T, Koch T, Litz RJ (2006). Modifying the baricity of local anesthetics for spinal anesthesia by temperature adjustment: model calculations. *Anesthesiology*.

[6] Lui ACP, Polis TZ, Cicutti NJ (1998). Densities of cerebrospinal fluid and spinal anaesthetic solutions in surgical patients at body temperature. *Canadian Journal of Anaesthesia*.

[7] Stienstra R, van Poorten JF (1988). The temperature of bupivacaine 0.5% affects the sensory level of spinal anesthesia. *Anesthesia and Analgesia*.

[8] Stienstra R, Gielen M, van Poorten F, Kroon JW (1989). Spinal anesthesia with plain bupivacaine 0.5%: regression of sensory and motor blockade with different temperatures of the anesthetic solution. *Anesthesia and Analgesia*.

[9] Arai YCP, Ueda W, Takimoto E, Manabe M (2006). The influence of hyperbaric bupivacaine temperature on the spread of spinal anesthesia. *Anesthesia and Analgesia*.

[10] Davis H, King WR (1954). Densities of cerebrospinal fluid of human beings. *Anesthesiology*.

[11] Hocking G, Wildsmith JAW (2004). Intrathecal drug spread. *British Journal of Anaesthesia*.

[12] Ririe DG, Walker FO, James RL, Butterworth J (2000). Effect of alkalinization of lidocaine on median nerve block. *British Journal of Anaesthesia*.

[13] Sinnott CJ, Garfield JM, Thalhammer JG, Strichartz GR (2000). Addition of sodium bicarbonate to lidocaine decreases the duration of peripheral nerve block in the rat. *Anesthesiology*.

[14] Powell MF (1987). Stability of lidocaine in aqueous solution: effect of temperature, pH, buffer, and metal ions on amide hydrolysis. *Pharmaceutical Research*.

[15] Kamaya H, Hayes JJ, Ueda I (1983). Dissociation constants of local anesthetics and their temperature dependence. *Anesthesia and Analgesia*.

[16] Stoner CD (2000). Inquiries into the nature of free energy and entropy in respect to biochemical thermodynamics. *Entropy*.

[17] Greene NM (1985). Distribution of local anesthetic solutions within the subarachnoid space. *Anesthesia and Analgesia*.

[18] Callesen T, Jarnvig I, Thage B, Krantz T, Christiansen C (1991). Influence of temperature of bupivacaine on spread of spinal analgesia. *Anaesthesia*.

[19] Foster RH, Markham A (2000). Levobupivacaine: a review of its pharmacology and use as a local anaesthetic. *Drugs*.

[20] Glaser C, Marhofer P, Zimpfer G (2002). Levobupivacaine versus racemic bupivacaine for spinal anesthesia. *Anesthesia and Analgesia*.

[21] Fattorini F, Ricci Z, Rocco A, Romano R, Pascarella MA, Pinto G (2006). Levobupivacaine versus racemic bupivacaine for spinal anaesthesia in orthopaedic major surgery. *Minerva Anestesiologica*.

[22] Richardson MG, Wissler RN (1996). Density of lumbar cerebrospinal fluid in pregnant and nonpregnant humans. *Anesthesiology*.

[23] Manara AR, Smith DC, Nixon C (1989). Sedation during spinal anaesthesia: a case for the routine administration of oxygen. *British Journal of Anaesthesia*.

